# “Ultrasmall” ZrO_2_ Nanoparticles: Disentangling Core and Surface Contributions to Structural and Electronic Properties through First-Principles Modeling

**DOI:** 10.1021/acsnanoscienceau.5c00088

**Published:** 2025-10-13

**Authors:** Ravikant Kumar, Assil Bouzid, Abid Berghout, Philippe Thomas, Olivier Masson

**Affiliations:** Institut de recherche sur la ceramiques (IRCER), CNRS UMR 7315, 27025 Universite de Limoges, Centre Europeen de la Ceramique, 12 rue Atlantis, Limoges 87068, France

**Keywords:** ZrO_2_ nanoparticles, ZrO_2_ polymorphs, core−shell structure, density functional theory, molecular dynamics, quantum confinement, surface passivation

## Abstract

We resort to first-principles molecular dynamics (FPMD) and density functional theory (DFT) calculations at the PBE and PBE0 levels of theory to examine the structure, stability, and electronic properties of zirconia nanoparticles (NPs) with diameters ranging from 0.9 to 2.0 nm. A procedure based on the use of water molecules and an appropriate MD thermal annealing cycle is developed to generate [ZrO_2_]_*n*_ models with different sizes (*n* = 14, 16, 43, 80, and 141) and different surface passivation states. It is shown that the rate of passivation has a significant influence on the NP structure and that NP models corresponding to saturated passivation exhibit the best structural characteristics, featuring close agreement with experimental atomic pair distribution functions (PDFs). It is also found that the Zr–O bond length varies as a function of the position of Zr and O atoms from the core to the surface of NPs, providing a descriptor capable of separating core and surface regions in ZrO_2_ NPs. A core–shell structure has been demonstrated for NP models as small as 1.3 nm, while for even smaller NPs, no separation between the core and shell is possible. For the largest NP models, the core atoms show local environments closer to the cubic phase of zirconia, while the local structure of atoms close to the surface shows a large similarity with the monoclinic phase. Finally, the study of electronic properties has shown that ZrO_2_ NPs exhibit very moderate quantum confinement effects. Moreover, the evolution of the band gap as a function of size does not correspond well with the *d* ^–2^ trend expected from the effective mass approximation model. These differences can only be partly attributed to the shell atoms, which induce a slight decrease in the band gap compared to the contribution of the core atoms.

## Graphical abstract

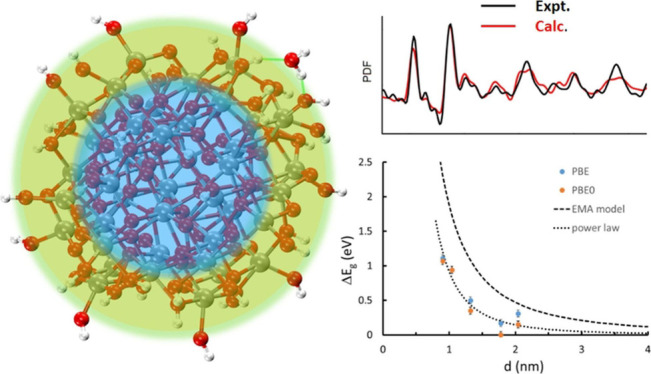

## 1 Introduction

Nanoparticles (NPs) of just a few nanometers in diameter have attracted a great deal of scientific and technological interest in recent decades, due to the unique properties and potential applications that emerge at such small dimensions.^1–13^ One of the most striking properties is certainly the tunability of the electronic and optical properties as a function of the particle size. It is well established that this behavior is mainly due to quantum confinement effects, caused by the small number of atoms in the particles.^2,6^

However, real NPs, especially metal oxide NPs, exhibit structural disorder depending on the degree of surface stabilization, controlled by the presence of impurities from the synthesis routes, so they generally cannot be treated as simple, small pieces of bulk material with a pure stoichiometric chemical composition. Recent studies suggest that NPs can exhibit a core–shell structure,^14^ unusual forms of structural disorder,^15–17^ and can undergo structural transformations, even reversible ones, in response to changes in the surface environment, rather than in the particle size.^18^ All of these types of structural deviation from the ideal bulk structure can significantly alter their properties. For example, it has been shown that low-coordinated surface Zr sites significantly lower the formation energy of oxygen vacancies and host trapped 4d electrons, which makes the NPs significantly more reducible than bulk zirconia^19^ or that defect-rich, dopant-free ZrO_2_ NPs exhibit room-temperature ferromagnetism, a property absent in the bulk material.^20^ Regarding electronic properties calculations, most of them are performed on NP models that retain the ideal structure of the bulk material and have surfaces fully passivated by more or less sophisticated methods.^21^ This is perfectly justifiable for the many quantum dots with zinc blende or wurtzite structures (e.g., CdSe, CdS, CdTe, InP, GaAs, InAs, ZnO, etc.), but maybe less in the case of metal oxide NPs with more complex and flexible structures.

Until now, the fine characterization of structural deviations of NPs from the ideal global structure is far from being solved^22–25^ as no experimental technique can provide unambiguous information for such small objects. Solving this problem requires additional information to be obtained through theoretical modeling methods.

This article focuses on the modeling of zirconia (ZrO_2_) NPs with a diameter of less than 3 nm. Zirconia is one of the leading wide-bandgap transition metal oxides, with applications in a wide range of technological fields due to its many excellent properties, such as high strength and hardness, chemical stability, and good ionic conductivity.^26–33^ It is promising in the field of nanoelectronics as a gate dielectric material in metal-oxide-semiconductor (MOS) transistors due to its high dielectric constant and its high band gaps compared to silicon.^28,34^ In addition, it possesses a complex polymorphism, with most of its polymorphs derived from the fluorite structure,^35–38^ and well-known structure-stabilization effects at the nanoscale.^39,40^ Finally, it turns out that ZrO_2_ NPs exhibit large structural deviations from the bulk structure.^41–43^

In a recent work,^17^ some of us have shown that proper modeling of the structural disorder present in real ZrO_2_ NPs requires incompletely saturated chemical bonds at the surface of the NP models. Indeed, complete surface passivation of ZrO_2_ NP models leads to unrealistic structures that too closely resemble the structure of bulk material. On the contrary, the absence of passivation leads to equally unrealistic, highly disordered, and amorphous-like structures. It has also been shown that the use of chemisorbed water molecules as model impurities is an effective and simple way to mimic possible stabilization mechanisms able to reproduce the real structure of ZrO_2_ NPs, although more complex molecules (e.g., alkoxide ligands) may be present in real systems.^44^

The aim of this paper is to address the following questions: Is there an efficient methodology leading to adequate surface passivation and realistic NP structures while avoiding prior knowledge of the required surface coverage? Is there a core–shell structure for such ultrasmall metal oxide NPs? If such a core–shell structure exists, what descriptor can distinguish core from shell regions, and what is the effect of such a structure on the electronic properties, particularly with regard to the band gap evolution and quantum confinement effect?

To address these questions, we resort to first-principles molecular dynamics (FPMD) and density functional theory (DFT) calculations at the PBE and PBE0 levels of theory to study the structure and electronic properties of zirconia NPs with diameters ranging from 0.9 to 2.0 nm. We use water molecules and appropriate MD thermal annealing to passivate dangling bonds on the surface of NPs up to a saturation rate. The quality of the models is assessed against experimental data via the X-ray total scattering pair distribution function (PDF) obtained from two samples of ZrO_2_ NPs synthesized by the sol–gel route in different conditions. The PDF method^45^ is particularly well-suited in this context as it is sensitive to local structural disorder. We use the Zr–O bond length as a descriptor for separating the core and surface regions of ZrO_2_ NPs and study the electronic properties as a function of the NP size. Finally, we discuss quantum confinement effects with regard to the core and surface structure and distortions present in the entire NPs.

## 2 Computational Details

All computations were performed on ZrO_2_ NPs with a maximum diameter of about 2 nm. Electronic structure calculations were conducted within the density functional theory formalism^46,47^ as implemented in the CP2K suite of programs.^48^ Unless explicitly specified, the Perdew–Burke–Ernzerhof (PBE)^49^ electron exchange and correlation functional was used for all the calculations, and the core–valence interactions were described by resorting to pseudopotentials of type Goedecker-Teter-Hutter.^50^ An atom-centered Triple-ζ valence with polarization (TZVP)^51^ basis set was employed to describe the orbitals, while an auxiliary plane-wave basis set was used for the expansion of the electron density. The Brillouin zone was sampled at the Γ point, and proper convergence of the total energy was achieved by setting the energy cutoff for the expansion of the plane waves to 400 Ry. In order to achieve calculated band gaps comparable to those of experimental measurements, we resorted to the PBE0 hybrid exchange and correlation functional. In this particular case, the fraction α of Hartree–Fock exchange added to the Hamiltonian was set to 28%, a value that allowed the achievement of calculated band gaps within the reported experimental bandgap range of cubic and monoclinic ZrO_2_. The atomistic models were placed in a periodic cubic simulation box with side lengths large enough to avoid interaction between periodic replicas, specifically of 20, 25, 30, 35, and 40 Å for [ZrO_2_]_14_, [ZrO_2_]_16_, [ZrO_2_]_43_, [ZrO_2_]_80_, and [ZrO_2_]_141_ NPs, respectively.

Structure relaxation and surface passivation of NPs were achieved using FPMD as implemented in CP2K within the Born–Oppenheimer (BOMD) scheme in the canonical ensemble (NVT) with a *t* = 1 fs time step for the integration of the equations of motion. A Nosé–Hoover thermostat chain ensured an optimal thermal control during the dynamics.^52^

The atomic PDFs of the atomistic models were calculated using an in-house software implementing an exact formalism^53^ to simulate the experimental PDFs obtained from X-ray total scattering measurements using a maximum scattering vector length (*Q*_max_) of 17.0 Å^–1^. They were averaged over the last 5 ps of the molecular dynamics trajectory.

## 3 Experimental Data

The quality of the structure of the atomistic models was assessed against experimental PDF data obtained from NP samples produced by soft chemistry and described in previous works.^17,54,55^ Two different synthesis routes, namely, hydrolytic (hereafter referred to as Exp1) and nonhydrolytic sol–gel routes (hereafter referred to as Exp2), were chosen in order to highlight the effect of the synthesis on the structure and modeling procedure of the NPs. The main characteristics of the two synthesis routes are briefly outlined here. The hydrolytic sol–gel route was based on zirconium n-propoxide, n-propanol, water, and acetylacetone (chelating agent) chemical system, whereas the nonhydrolytic route was based on zirconium isopropoxide and anisole system in alkaline conditions. With the first method, the water molecules present in the reaction mixture correspond more closely to the surface passivation model used in this work, while the second is recognized as a very effective method for obtaining small, spherical, monodisperse, and nonaggregated particles. Figure 1 shows the experimental PDFs of the two samples measured with a laboratory X-ray total scattering setup (details are given in refs 17, 54 ).

**Figure 1:**
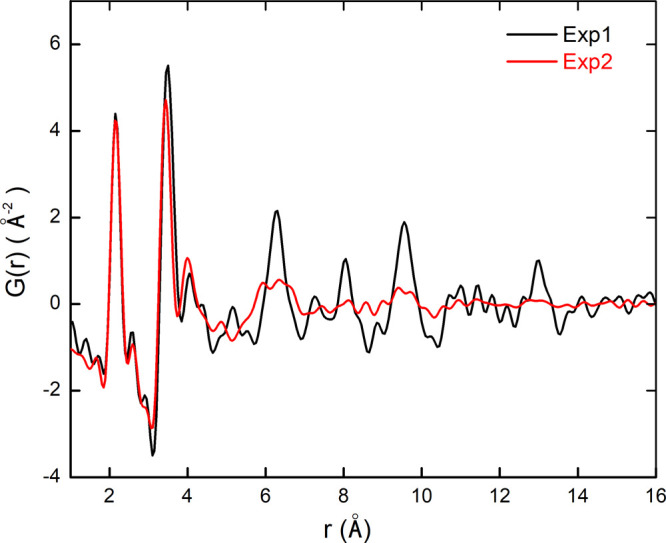
Experimental PDFs of sample Exp1 (black) and sample Exp2 (red). Data reproduced from refs. 17, 54 .

It can be seen that the dampening of the PDFs is very abrupt (see also the PDF plot over a wider range in Figure S1 in the Supporting Information), indicating that both synthesis methods produced zirconia NPs with very small correlation lengths. These latter can be visually estimated at around 1.8 and 1.3 nm for Exp1 and Exp2, respectively. The first peak at about 2.15 Å corresponds to Zr–O bond lengths. The peak corresponding to the shortest O–O distance at 2.70 Å is barely visible due to the low scattering power of oxygen atoms (with respect to Zr atoms) and merges with the oscillations induced by the finite *Q*_max_ value used experimentally (17.0 Å^–1^). The *Q*_max_ effect should, however, be very limited, given the relatively high positional disorder of the Zr and O atoms, which induces a significant broadening of the PDF peaks. As illustrated in Figure S2 in the Supporting Information, which compares the PDFs calculated from the [ZrO_2_]_43_ NP model with and without the termination effect, these almost overlap, indicating that the *Q*_max_ effect is probably negligible beyond the very first peak. The shortest Zr–Zr distances correspond to the most intense peak at around 3.5 Å for Exp1, and slightly less for Exp2. The subsequent peaks, which lie between 3.9 and 4.7 Å, are mainly correlated with Zr–O distances. Interestingly, this region of low r values cannot be fully interpreted on the basis of the known ZrO_2_ polymorphs, indicating the presence of structural disorder within the NPs.^17^ This is illustrated in Figure S3 of the Supporting Information, where the PDF of sample Exp1 is compared to those calculated from the zirconia polymorphs deriving from the fluorite structure.

## 4 Generation of Realistic NP Models

### 4.1 Methodology

We considered the cubic structure of ZrO_2_ (i.e., the most symmetrical) to build the initial configurations of the NPs models. In practice, we cut a sphere of radius *r* around a central atom, either Zr or O, and adjusted the number of atoms at the NP surface to achieve stoichiometric [ZrO_2_]_*n*_ models (with *n* an integer). To investigate the size effect on the properties of ZrO_2_ NPs, five models were generated, namely [ZrO_2_]_*n*_ with *n* = 14, 16, 43, 80, and 141, corresponding to diameters of 0.9, 1.04, 1.32, 1.78, and 2.04 nm, respectively. NPs obtained in this way have dangling bonds and undercoordinated atoms on their surface, which can lead to excessively disordered structures and unrealistic electronic properties. A surface passivation procedure was therefore applied. We used chemisorbed water molecules as this is an effective and simple way to remove dangling bonds and complete the coordination shells of Zr atoms, although more complex molecules (e.g., alkoxide ligands in the case of nonhydrolytic sol–gel routes) may be present in real systems. It is known that the nature of the ligands (ionic vs nonionic, short vs long chain) can significantly alter the NP structure.^56^ However, the exact way ligands modify the structure of NPs involves complex mechanisms, tightly bound to the nature of the ligand, thereby making it hard to describe with a general model. Within this context, modeling surface passivation with water molecules, although simplistic, is a good compromise between model complexity and efficiency. It should also be pointed out that water passivation of NP models can significantly influence the calculated properties. For example, it has been observed that ZrO_2_ NPs can exhibit marked ferromagnetic behavior,^20^ and that this property can be theoretically explained by the presence of electrons trapped on low-coordination surface Zr ions present in unpassivated NP models.^57^ In our study, passivation by water molecules, even incomplete, neutralizes most of the low-coordination sites and thus could significantly reduce or even eliminate magnetism.

Instead of manually dissociating water molecules on given surface sites, we followed a scheme that avoids prior knowledge of the required surface coverage. For this purpose, water molecules (with the number depending on the NP size) were spread out at a distance of 2.2 Å from the surface Zr atoms, and their orientation was adjusted to keep them as far apart as possible, thereby ensuring an almost uniform distribution. The maximum number of water molecules used was set equal to the number of surface Zr atoms with less than 6 neighbors. This arbitrary value proved adequate for all the models studied and led to 8, 12, 24, 26, and 54 water molecules for the [ZrO_2_]_14_, [ZrO_2_]_16_, [ZrO_2_]_43_, [ZrO_2_]_80_, and [ZrO_2_]_141_ NPs, respectively. Note that we also produced models with fewer water molecules to test the effect of a low passivation rate. These initial configurations were first relaxed at 0 K, leading to the spontaneous dissociation of a few water molecules at the NP surface (with H atoms bonding to the O surface atoms and OH groups bonding to the Zr surface atoms). FPMD was then applied to overcome energy barriers, leading to relaxed structures with optimal passivation rates at near-ambient conditions. Three different thermal annealing treatments were tested in order to explore the efficiency of the annealing procedure for the final NP structure. They are represented in Figure 2. In the first one, the system is annealed at 300 K for 25 ps (thermal treatment 1, called hereafter TT1). In the second, after 5 ps at 300 K, the system is brought to 550 K and annealed for a duration of 25 ps (thermal treatment 2, called hereafter TT2). The third thermal treatment (TT3) consists of a full annealing cycle as follows: 5 ps at 300 K, 5 ps at 550 K, 25 ps at 750 K, 4 ps at 550, and 10 ps at 300 K.

**Figure 2:**
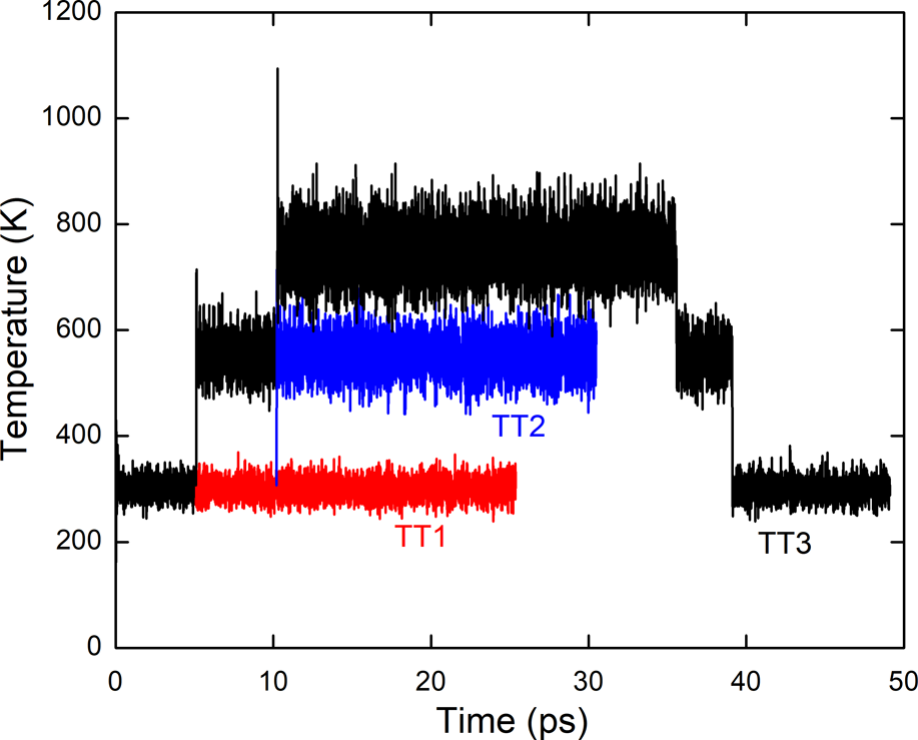
Temperature evolution during MD simulation corresponding to the three different thermal treatments: TT1 (red), TT2 (blue), and TT3 (black).

We first illustrate the impact of thermal treatment with the [ZrO_2_]_43_ model (1.32 nm in diameter). The initial configuration, with 24 H_2_O, and the configuration after MD thermal treatment TT3, are shown in Figure 3a,b, respectively. After the MD run, most of the water molecules are chemisorbed (dissociated into H and OH), and the few undissociated molecules keep close to the surface, forming H-bonds. We can also note the appearance of significant positional disorder in the MD-annealed structure, which nevertheless retains crystalline character in view of the atomic planes, which are still clearly visible.

**Figure 3:**
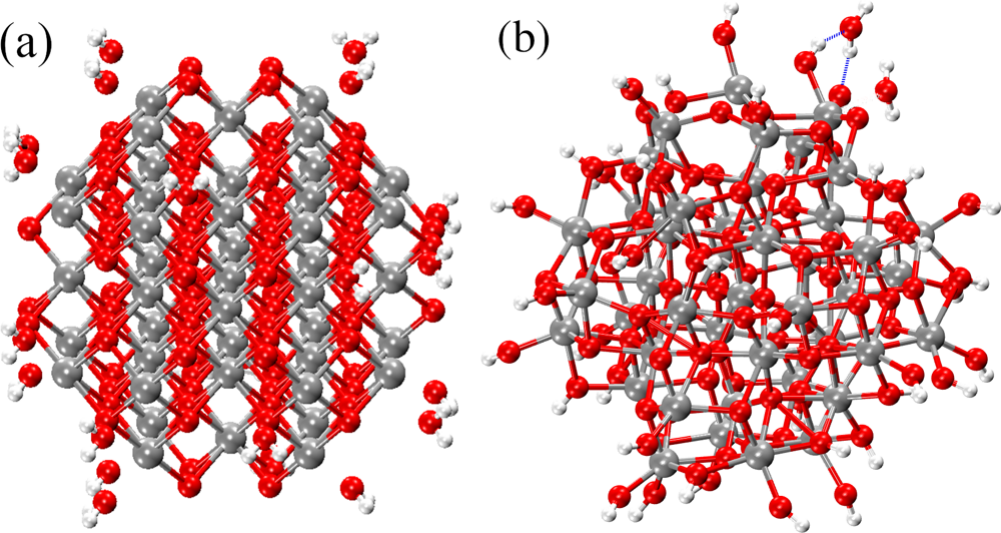
(a) Initial structure of [ZrO_2_]_43_ NP covered with 24 water molecules and (b) final structure obtained after MD thermal treatment TT3. Zirconium, oxygen, and hydrogen atoms are in silver, red, and white, respectively. The blue-dotted line shows the hydrogen bonds.

Figure 4 shows the evolution of the number of chemisorbed H_2_O molecules over time. Irrespective of the thermal cycle, the number of dissociated molecules reaches a plateau before the end of the simulation, typical of saturation at the considered temperature. Comparing the three thermal treatments, annealing at 300 K (TT1) leads to the dissociation of 20 water molecules, heating to 550 K (TT2) permits the chemisorption of a slightly larger number of molecules (23), while a full annealing cycle (TT3) leads to a similar number of chemisorbed molecules (fluctuating around 22 ± 1).

**Figure 4:**
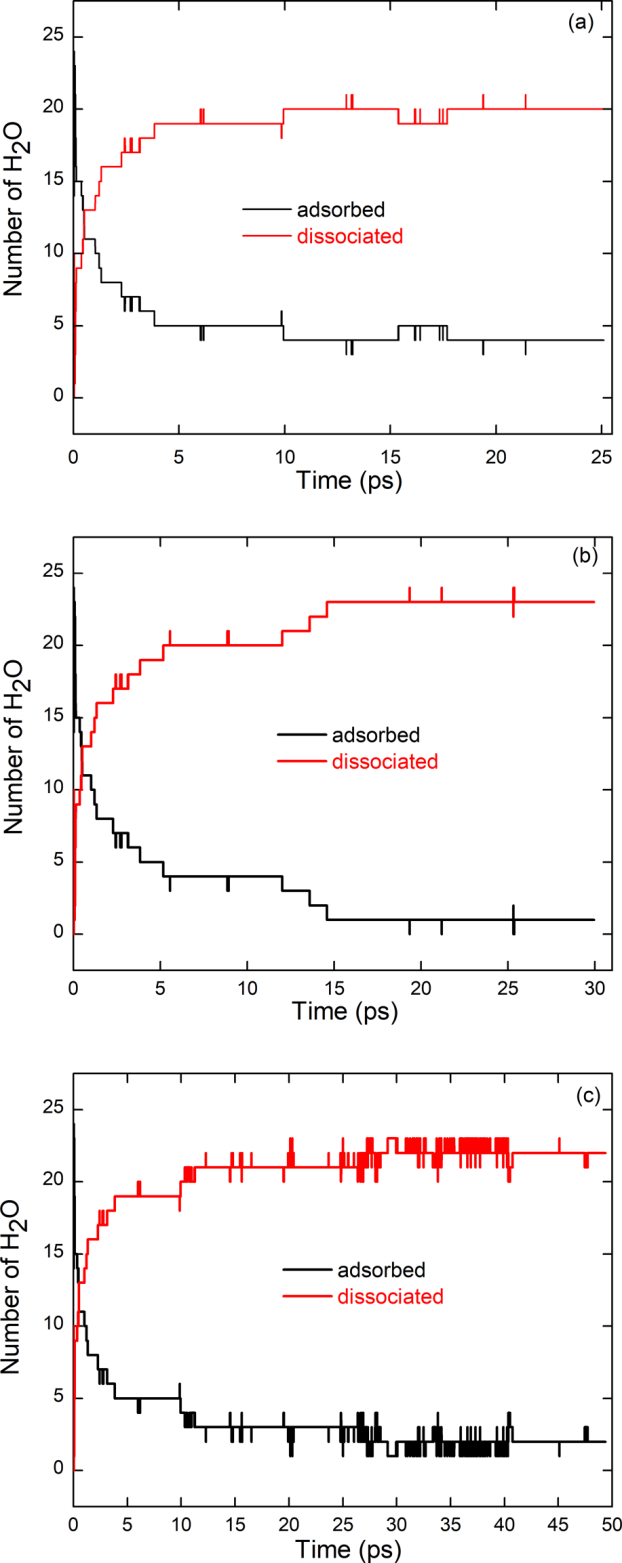
Number of chemisorbed (i.e., dissociated) and physisorbed H_2_O molecules at the surface of [ZrO_2_]_43_ NP during MD thermal treatments TT1 (a), TT2 (b), and TT3 (c).

The evolution of the water molecule dissociation rate as a function of the temperature of the TT3 heat treatment is shown in Figure 5a. It can be seen that for all the NP models, the number of dissociated water molecules first increases with temperature and then remains constant after reaching 750 K, even when the temperature is reduced to 300 K. In addition, a 100% passivation is achieved for the smallest *n* = 14 and *n* = 16 NPs. In Figure 5b, the chemisorption rate is depicted as a function of NP size after the thermal treatment TT3. We observe a decrease in this rate with increasing NPs size, with a minimum value of 85% reached for the largest NP.

**Figure 5:**
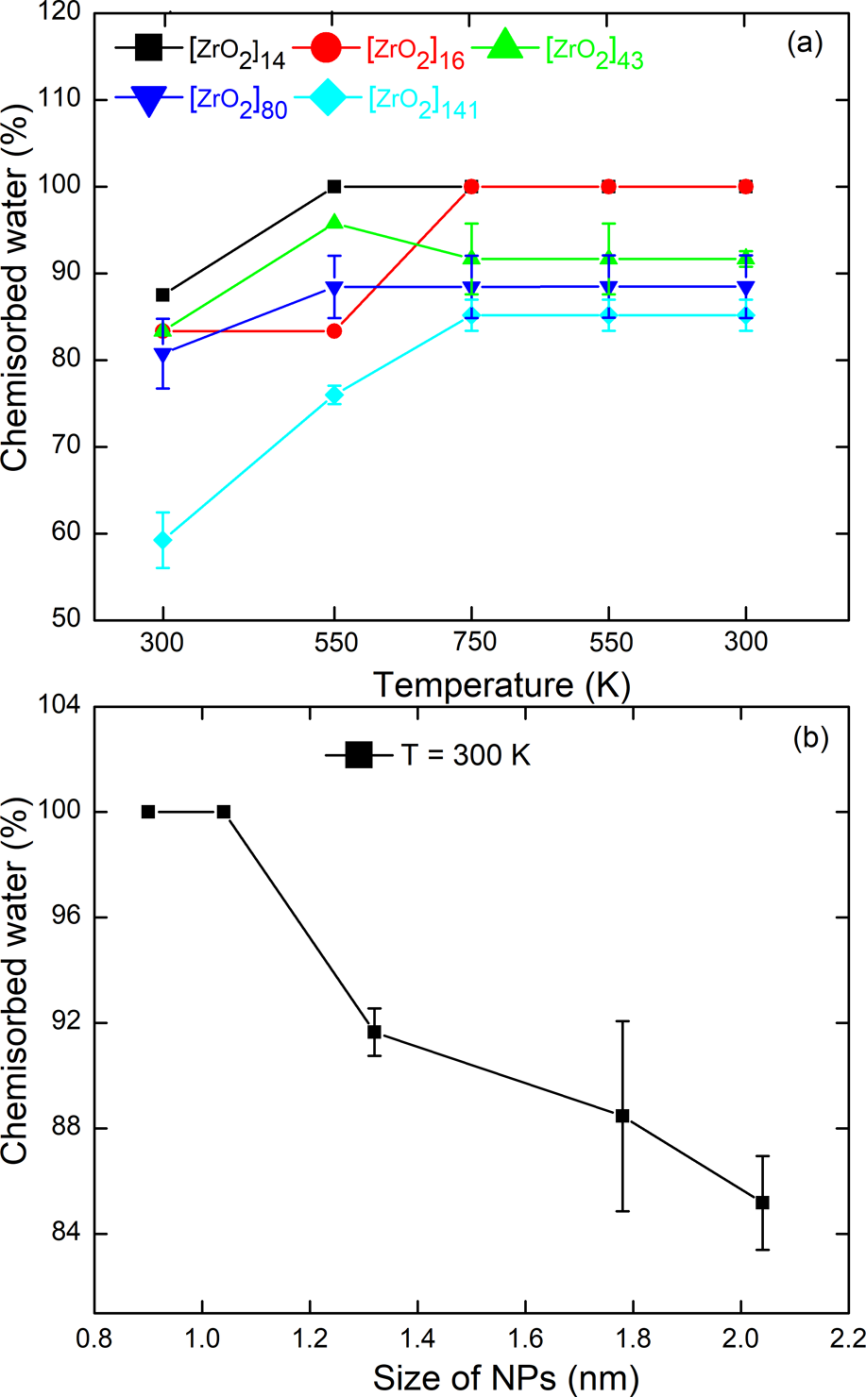
Evolution of the rate of chemisorbed water molecules as a function of temperature (a) during thermal treatment of TT3 and NP size (b) after quenching at *T* = 300 K.

All of these results highlight the efficiency of using molecular dynamics in achieving surface passivation without any prior knowledge of the required surface coverage.

### 4.2 Assessment of the NP Models

We first assessed the quality of the obtained models using the [ZrO_2_]_43_ NP with the highest passivation rate (92%). Figure 6 compares the PDFs calculated over the last 5 ps of the MD trajectory after the TT1, TT2, and TT3 thermal treatments with the two experimental ones. Inspection of Figure 6a reveals that the models are overall consistent with sample Exp1, with TT3 giving the best agreement. The first peaks are fairly well reproduced in terms of position, intensity, and width, especially in the range from 3 to 5 Å. The two close peaks at 4.0 and 4.4 Å are perfectly reproduced in the case of the TT3 model only. A similar trend is observed in the region from 7.1 to 8.5 Å, where the TT3 model better reproduces the two experimental peaks. For larger distances, no heat treatment is able to reproduce the experimental data because of the limited size of the [ZrO_2_]_43_ model.

**Figure 6:**
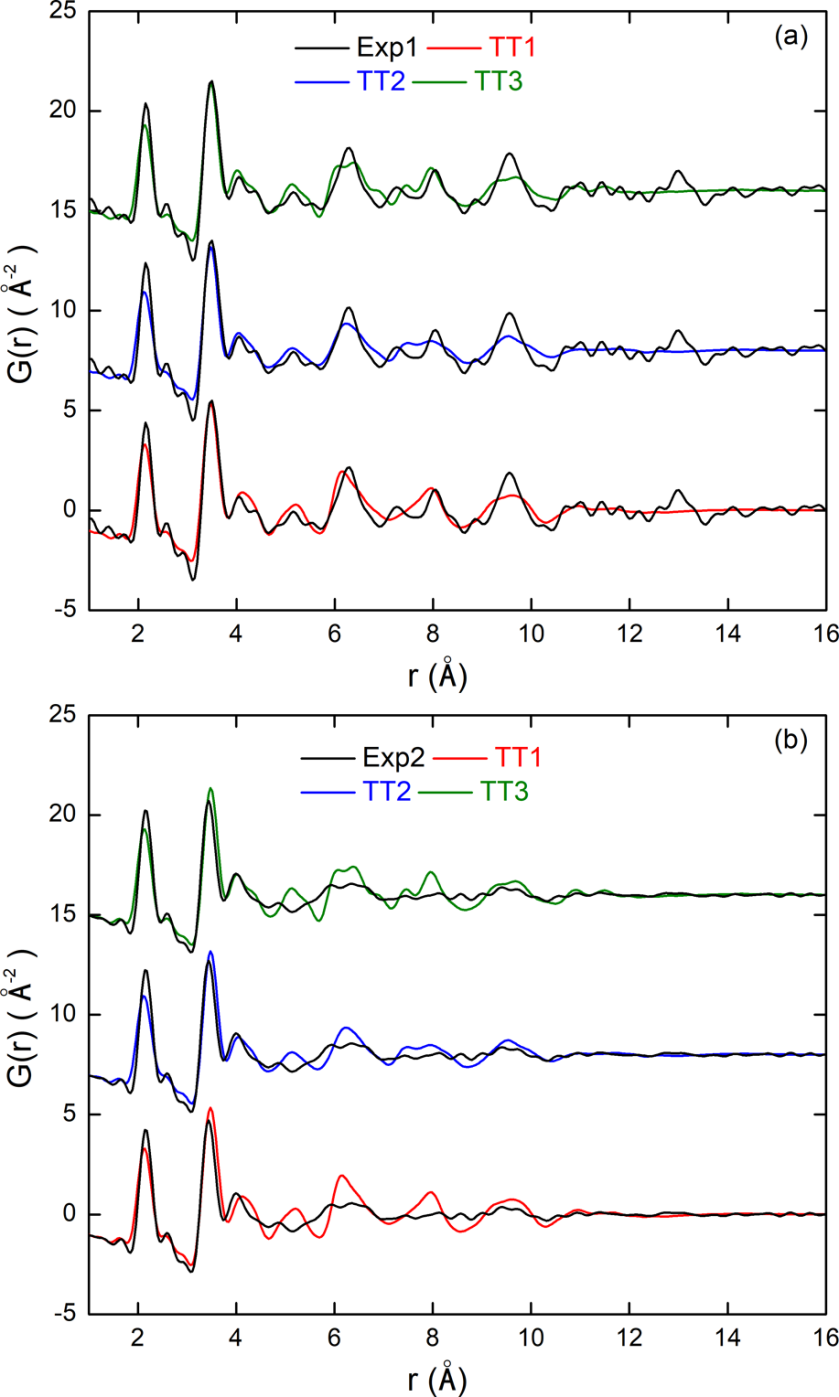
PDFs calculated from [ZrO_2_]_43_ NP models obtained after TT1, TT2, and TT3 heat treatments, compared with experimental PDFs of samples Exp1 (a) and Exp2 (b).

Figure 6b compares the calculated PDFs of the [ZrO_2_]_43_ models with the data from sample Exp2. All of the models reproduce the experimental PDF fairly accurately for values of *r* below 5 Å and above 9 Å, with the TT3 model being slightly better in the region around 4 Å. However, in the intermediate region, all three models give peaks that are better defined than those measured experimentally, suggesting that they still lack disorder.

Overall, it appears that the TT3 treatment, implementing a full annealing cycle, produces the best models of relaxed structures at room temperature, capable of reproducing the fine details of both experimental PDFs.

Besides the thermal treatment and NP size, the surface coverage is another important factor that can affect the structure of the simulated NPs. In the above analysis, we considered only NP models whose surface coverage corresponds to saturation. We now focus on the effect of a lower stabilization effect. To this end, we also built models based on the [ZrO_2_]_43_ and [ZrO_2_]_141_ NPs with under-saturated surface coverage of 16.7 and 33.3% and annealed them using the TT3 treatment. These passivation rates correspond, respectively, to 4 and 8 chemisorbed H_2_O molecules for the [ZrO_2_]_43_ model and 9 and 18 molecules for the [ZrO_2_]_141_ model. The PDFs of the corresponding models for [ZrO_2_]_43_ are presented in Figure 7a,b and are compared with the experimental data. We remark that as the passivation rate decreases, the two intense peaks (corresponding to Zr–O and Zr–Zr distances) shift their positions toward lower distances compared to both experimental PDFs. This trend can be explained by the increase in the fraction of dangling bonds at the surface of NPs, leading to a decrease in the Zr coordination number and consequently a shortening of the Zr–O bonds and contraction of the Zr–O–Zr bridges. Focusing on Exp1, we find that the PDF of the model with the saturated passivation rate (reproduced from Figure 6 for comparison) shows the best agreement with the experimental data. In contrast, for Exp2, under-passivated models seem to be the best compromise, as they better reproduce the flat, broad peaks of the experimental PDF in the *r* region above 5 Å, while maintaining good agreement in the low *r* region.

**Figure 7:**
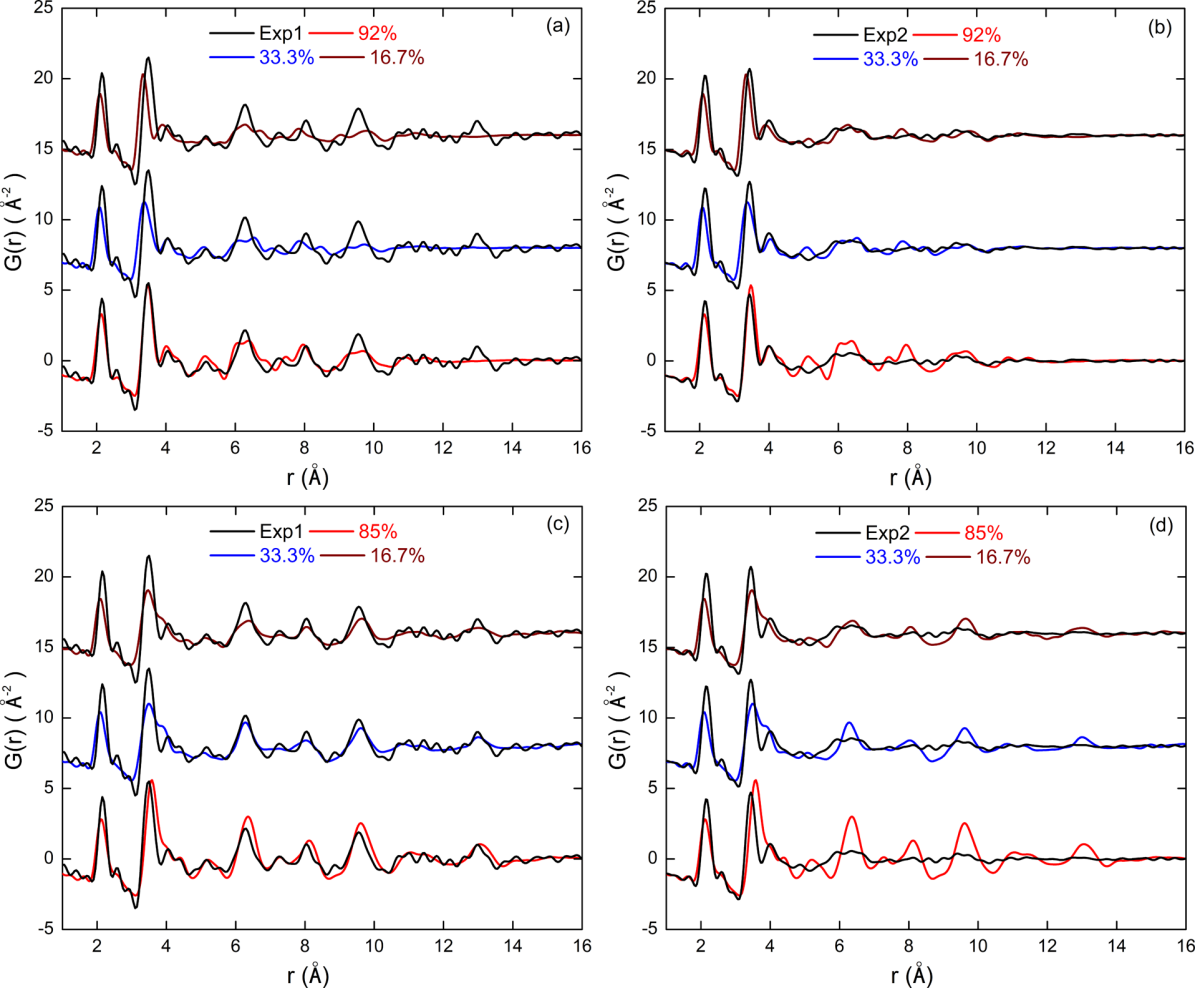
PDFs calculated from the under-passivated (16.7 and 33.3%) NP models [ZrO_2_]_43_ (a, b) and [ZrO_2_]_141_ (c, d) obtained with thermal treatment TT3, and compared with the experimental PDFs of samples Exp1 (a, c) and Exp2 (b, d). The calculated PDFs of the saturation passivated ZrO_2_]_43_ (92%) and [ZrO_2_]_141_ (85%) NP models are also plotted for comparison.

In the case of the [ZrO_2_]_141_ NPs (Figure 7c,d), the best agreement with Exp1 is also obtained for the saturation passivated models. However, although this model reproduces the peaks of the PDF fairly well for intermediate and larger values of *r*, it does not capture the details of the low values of the *r* region as well as the [ZrO_2_]_43_ model does. For sample Exp2, none of the considered [ZrO_2_]_141_ models can reproduce the experimental PDF, certainly due to an excessively large model size.

Overall, these results suggest that the [ZrO_2_]_43_ model produced following the full thermal annealing cycle TT3 is able to capture the details of both Exp1 and Exp2 samples by varying its surface passivation rate from 92 to 16.7%, respectively. This observation seems to be consistent with the synthesis protocols used in Exp1 and Exp2. Indeed, we expect a greater passivation effect under hydrolytic conditions, as water molecules can more easily bind to metal oxide surface sites than larger organic molecules present in a nonhydrolytic reaction medium. In addition, these results show that achieving an accurate structural model of metal-oxide NPs requires an assessment of the particle size, the surface passivation rate, and the MD thermal treatment.

## 5 Results and Discussions

### 5.1 Structure Evolution as a Function of the NP Size

#### 5.1.1 The Overall Structure of the NP Models

In order to investigate the NP structure evolution as a function of their size, we computed the partial PDF on the NP models produced through a full thermal annealing cycle (TT3) with a saturated passivation rate. The obtained results for the [ZrO_2_]_*n*_ models with *n* = 14, 16, 43, 80, and 141 are shown in Figure 8. By looking at the Zr–O partial PDF (Figure 8a), regardless of the NP size, we see the first intense peak occurring at around 2.12 Å, characteristic of the Zr–O chemical bond length. The second and third peaks, located at distances between 3 and 6 Å, are well resolved and clearly visible for the larger NP models and become broader and less intense as the size of the NP decreases. In the case of [ZrO_2_]_14_ and [ZrO_2_]_16_ NPs, these peaks are particularly spread over the range of *r* values considered. These differences observed with decreasing NP size are very similar to those between the Zr–O partial PDFs of cubic and monoclinic zirconia (see, for example, the partial PDFs of the cubic and monoclinic polymorphs in Figure 10 of the following section). This suggests that smaller NPs tend to have a more monoclinic-like structure. For higher *r* values, the peaks are dampened due to the reduction in NP size, but oscillations are still visible up to *r* values close to the NP diameter, reflecting the crystalline nature of the NP.

**Figure 8:**
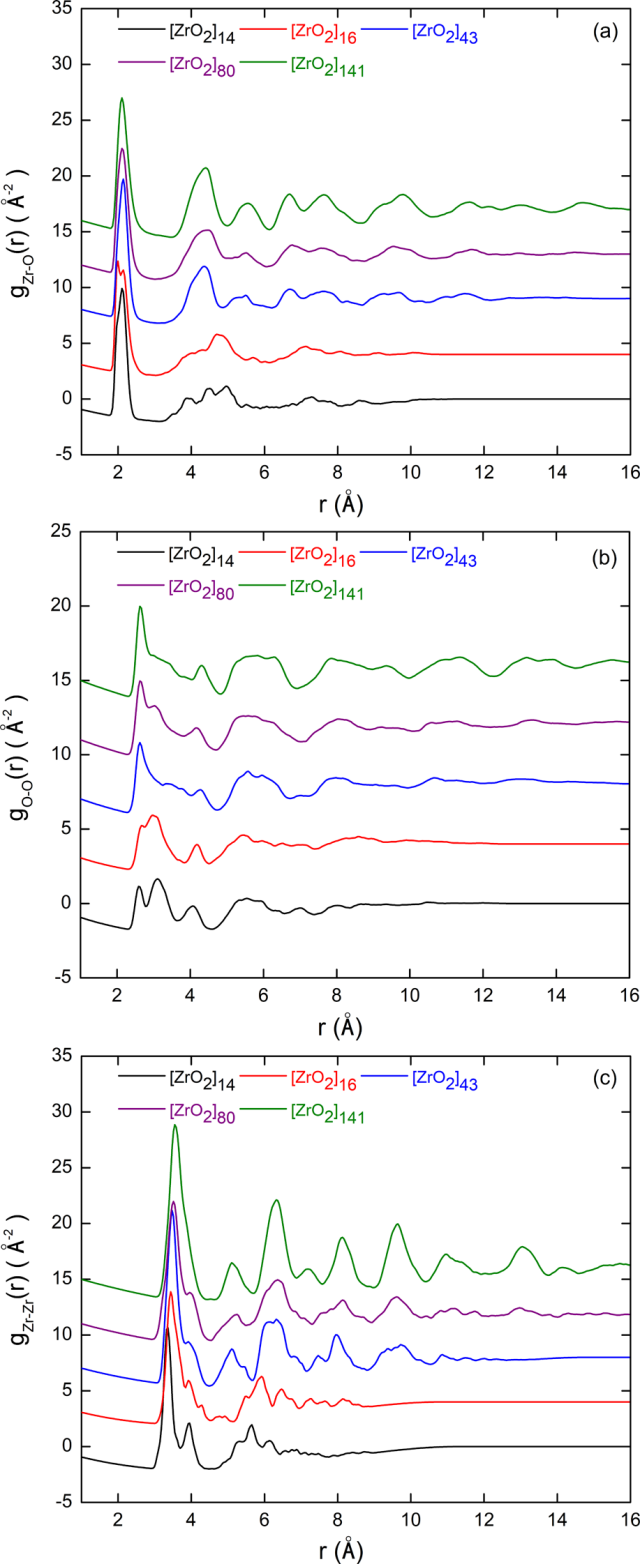
Zr–O (a), O–O (b), and Zr–Zr (c) PDFs calculated for the different NP models.

Focusing now on the partial PDFs of the O–O group (Figure 8b), we can see for the largest NP an intense peak at 2.7 Å, followed by a shoulder at 3.3 Å. As the size of the NPs decreases, the first peak shifts slightly toward lower *r* values and the shoulder turns into a well-separated intense peak. At the same time, the third peak at around 4 Å, clearly visible for all NP sizes, shifts slightly toward higher *r* values. Overall, the partial PDF of the smallest NP more closely resembles that of the monoclinic polymorph, but the trend observed in the evolution with NP size of the shape of the Zr–O partials, from cubic to monoclinic, is less evident here. The Zr–Zr partial PDFs (Figure 8c) show the most significant changes as a function of the NP size. For the largest model, a single broad peak centered around 3.6 Å is present, followed by 3 peaks at about 5.1, 6.3, and 7.3 Å. As the NP size decreases, the first peak shifts to lower *r* values and splits into two, leading to an intense peak at 3.3 Å followed by a smaller one at 3.9 Å for the smallest NP. At the same time, the next three peaks change in position and intensity to form a flatter shape made of 4 bumps at around 5.2, 5.7, 6.2, and 6.5 Å for the smallest model. As with the Zr–O partial, this evolution suggests a transition from a more cubic to a more monoclinic-like structure with decreasing NP size.

The above analysis shows that there is a strong correlation between the size of NPs and their structure; in particular, the smaller the size, the closer the structure is to the monoclinic polymorph. Given the high proportion of surface atoms in NPs, this opens the question of the contribution of surface and near-surface atoms to the overall structure and electronic properties of NPs, as well as the question of the presence of a boundary between near-surface atoms and core atoms for such small particles.

### 5.2 Disentangling the Core and Shell Structure Contributions

In order to establish whether there is a boundary between the atoms belonging to the core and those close to the surface, we used the length of the Zr–O bond as a structural fingerprint. This choice was motivated by the sensitivity of this descriptor to the coordination of the Zr atom. Indeed, Zr atoms on the NP surface are expected to have bonds with terminal or under-coordinated O atoms, which should be slightly shorter than those in the reference bulk material. In practice, we calculated the average Zr–O bond length by considering only the atoms inside a shell of variable radius *r* and thickness 3 Å centered on the central atom of the NP model. The shell radius was increased from small to large values, providing the average evolution of the Zr–O bond length from the center of the NP to its surface. All of the calculations were averaged over the last 5 ps of the considered trajectory. The results are provided in Figure 9.

**Figure 9:**
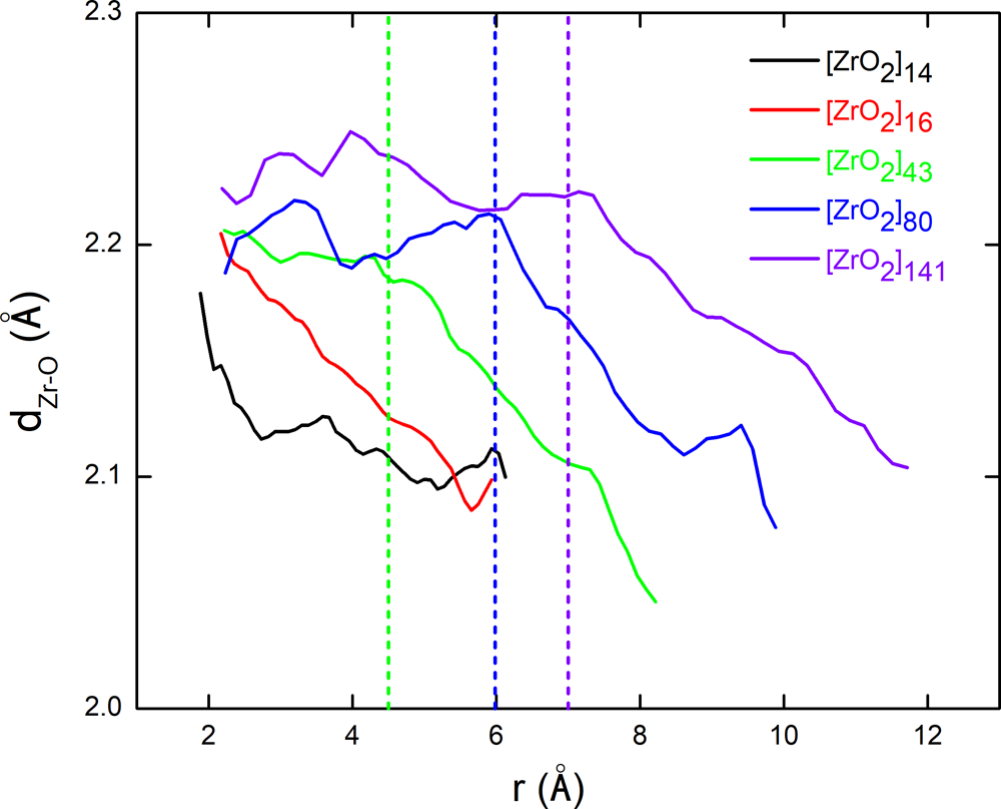
Evolution of the average Zr–O bond length, calculated from atoms inside a shell of variable radius *r* and thickness 3 Å centered on the central atom of the NP model as a function of *r*, for the different NP models.

For the two smallest NPs, the average Zr–O distance immediately decays beyond the coordination sphere of the central atom and reaches a value around 2.1 Å at the surface of the NP. This behavior can be explained by the fact that for such small NPs, the surface has such a strong influence on the overall NP structure that it is not possible to make a distinction between the core atoms and the surface or near-surface atoms. On the other hand, for the larger models, the average Zr–O bond length is initially almost constant, with a value close to 2.2 Å, before decreasing toward values on the order of 2.1 Å. Threshold values around 4.5, 6.0, and 7.0 Å are found for the [ZrO_2_]_*n*_ models with *n* = 43, 80, and 141, respectively. These values delimit the regions from which surface effects start to be important, thus affecting the average Zr–O bond length. This result can be interpreted as the formation of a core–shell structure. The shell thickness ranges from about 2 to 3 Å when going from [ZrO_2_]_43_ to [ZrO_2_]_141_ model, corresponding to about 70–80% of atomic fraction. This roughly represents one to two metal-oxide layers.

#### 5.2.1 Structural Properties of the Core and Shell Atoms

We now use the core–shell thresholds established in the previous section and investigate the structural features of each region of the NP. To this end, we computed the Zr–O, O–O, and Zr–Zr distances for atoms belonging to the core or to the shell of the NPs, and compared them to the partial PDFs calculated from the cubic and monoclinic ZrO_2_ polymorphs. The results are shown in Figure 10.

**Figure 10:**
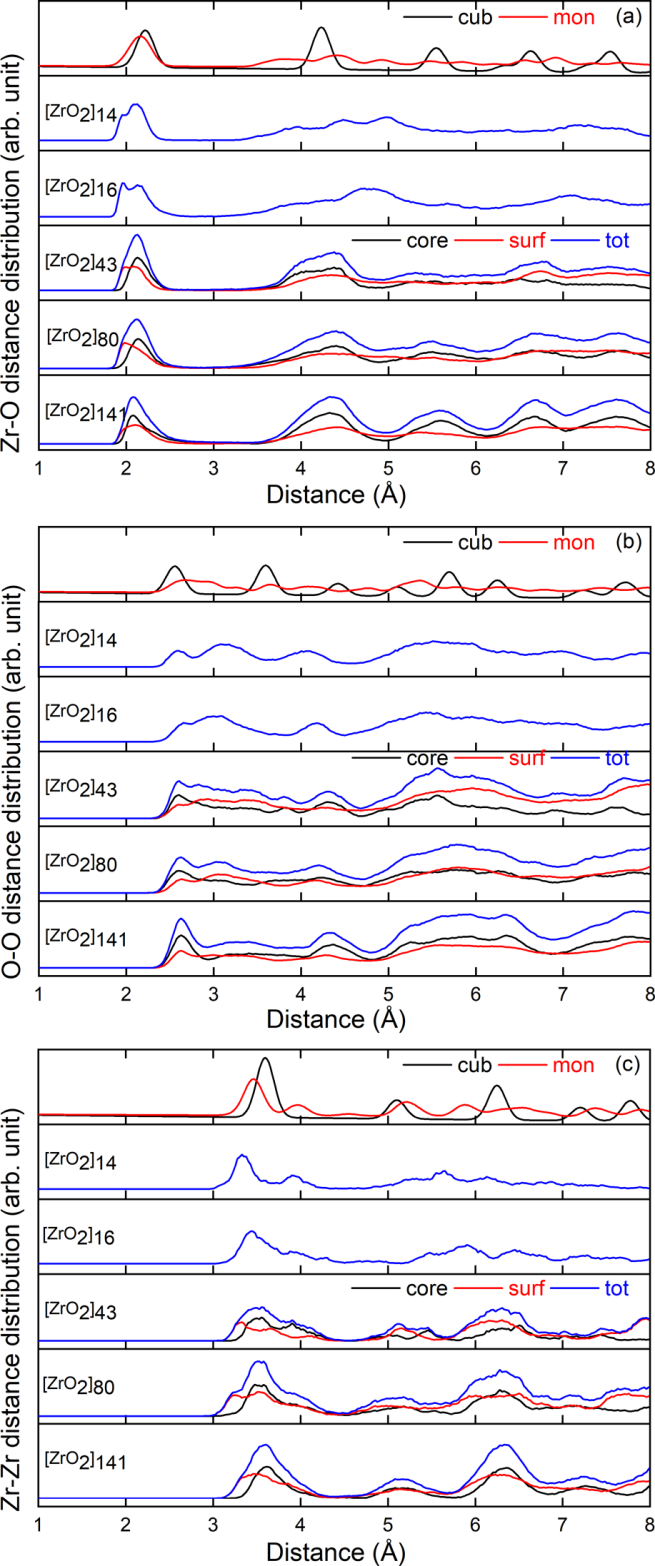
Distribution of the Zr–O (a), O–O (b), and Zr–Zr (c) distances found in the different NP models and broken down into core (black) and surface (red) contributions. The calculated partial PDFs of cubic and monoclinic zirconia polymorphs are also shown (upper curves) for comparison.

In the case of the smallest models, *n* = 14 and 16, the distributions were calculated considering all the atoms in the NP models, as no core-surface threshold could be established. The results obtained for the Zr–O, O–O, and Zr–Zr distances in these systems show a high similarity with the corresponding partial PDFs of the monoclinic structure in terms of peak positions and widths, which is in line with the discussion of previous sections.

For larger NPs, Figure 10a shows that the first peak of the Zr–O distances is made up of two distinct contributions. The first comes from atoms belonging to the NP core and is centered around 2.14, 2.14, and 2.08 Å for the [ZrO_2_]_43_, [ZrO_2_]_80_, and [ZrO_2_]_141_ models, respectively. These peaks occur at a slightly shorter distance than the Zr–O distance in the cubic phase of zirconia. This is particularly evident for the [ZrO_2_]_141_ model. Observing the shape of the Zr–O bond length distribution profile, we note an asymmetry toward large r values with a small shoulder at around 2.30 Å. The average value of the Zr–O bond length weighted over the entire first peak (from 0 to 3 Å) is 2.22 Å. This peak shape, therefore, suggests local deformation of the Zr atom environment with both shortening of some bonds (2.08 Å) and lengthening of others (2.30 Å). The second contribution comes from the shell atoms. The peaks are broader and are located at shorter distances, recalling those of the monoclinic structure. Similarly, for larger distances, the distributions calculated from the shell atoms are flatter than those calculated from the core atoms, which is also reminiscent of a monoclinic distortion. In the case of O–O correlations, the atoms belonging to the NP core generate a well-defined first peak, located at 2.60, 2.62, and 2.64 Å for [ZrO_2_]_43_, [ZrO_2_]_80_, and [ZrO_2_]_141_ models, respectively, corresponding better to what is observed with the cubic structure. For the shell atoms, a much broader and almost flat peak is observed in the range of 2.4–3.6 Å, similar to that observed in the monoclinic phase. Finally, the Zr–Zr correlations in the core of the NP feature first distances of 3.49, 3.54, and 3.57 Å in the case of [ZrO_2_]_43_, [ZrO_2_]_80_, and [ZrO_2_]_141_, respectively, in close agreement with those reported in cubic ZrO_2_. Those on the surface of the NP show a slightly shorter distance in better agreement with the monoclinic zirconia polymorph. The second peak at 4 Å, typical of the monoclinic structure, is also present for most of the NP models. Interestingly, this peak is more pronounced in the case of the small NPs and tends to merge with the main Zr–Zr peak as the NP size increases. In addition, both the surface and core Zr atoms contribute to this peak irrespective of the NP size. In the case of the largest [ZrO_2_]_141_ model, we note that all of the peaks due to the core atoms reproduce very well the Zr–Zr partial PDF of the cubic phase, while those induced by the shell atoms are similar to the partial PDF of the monoclinic phase, albeit with substantial broadening.

This analysis demonstrates the effectiveness of using the Zr–O bond length to distinguish the core and surface regions of the ZrO_2_ NPs. In addition, by focusing on the distribution of the Zr–O, O–O and Zr–Zr distances, it turns out that the core of the largest NPs shows a strong similarity to the cubic phase, while the surface of the NPs tends to be closer to the monoclinic phase. Such a finding highlights the importance of surface reconstruction of metal-oxide NPs, where a simple ideal NP model will fail in explaining the overall structure of the NP.

### 5.3 Electronic Properties

#### 5.3.1 Density of States of Whole NPs

So far, we have shown that the generation of good NP models, consistent with experimental data, requires a proper surface passivation procedure as well as adequate thermal treatment. This allows the surface atoms to reorganize and relax, leading to larger NPs and the formation of a core–shell structure with different structural fingerprints. To study the effect of this particular structure on the electronic properties, we calculated, at different levels of theory, the total (TDOS) and partial (PDOS) densities of states and bandgap widths of NP models and compared them with those of cubic and monoclinic polymorphs of zirconia. For each NP model, TDOS and PDOS calculations were averaged over 10 snapshots taken from the last 5 ps of the MD trajectory.

Using the PBE exchange and correlation functional, the calculated band gap values of the cubic and monoclinic polymorphs are 3.26 and 3.58 eV. These values are well below the reported experimental values, which range from 5 to 6 eV depending on the synthesis method and the type of sample.^58–60^ This discrepancy is a well-known problem of the GGA family of DFT functionals, which can be corrected by resorting to a higher level of theory, in particular, using hybrid functionals. The use of PBE0 ^61,62^ hybrid functional allowed obtaining band gap values of 5.91 and 6.09 eV for the cubic and monoclinic polymorphs, respectively, falling within the reported experimental band gap range. Accordingly, both PBE and PBE0 were used for electronic structure calculations of the NP models. The TDOS and PDOS of monoclinic and cubic zirconia calculated using PBE and PBE0 are shown in Figures 11 and 12. We can see that the two functionals give very similar DOS profiles. In addition, the DOS profiles of the cubic and monoclinic polymorphs show relatively similar profiles. Slight shifts toward higher energies are observed for the monoclinic phase. In particular, the conduction band of the monoclinic phase shifts to higher energy values than that of the cubic phase, resulting in a larger energy band gap. The PDOS plots show that for both phases, the valence band is mainly made up of the p-orbitals of O atoms, and that the Zr atoms' d-orbitals are mainly responsible for the conduction band. These results are consistent with the HOMO and LUMO orbitals shown in Figure S4 in the Supporting Information.

**Figure 11:**
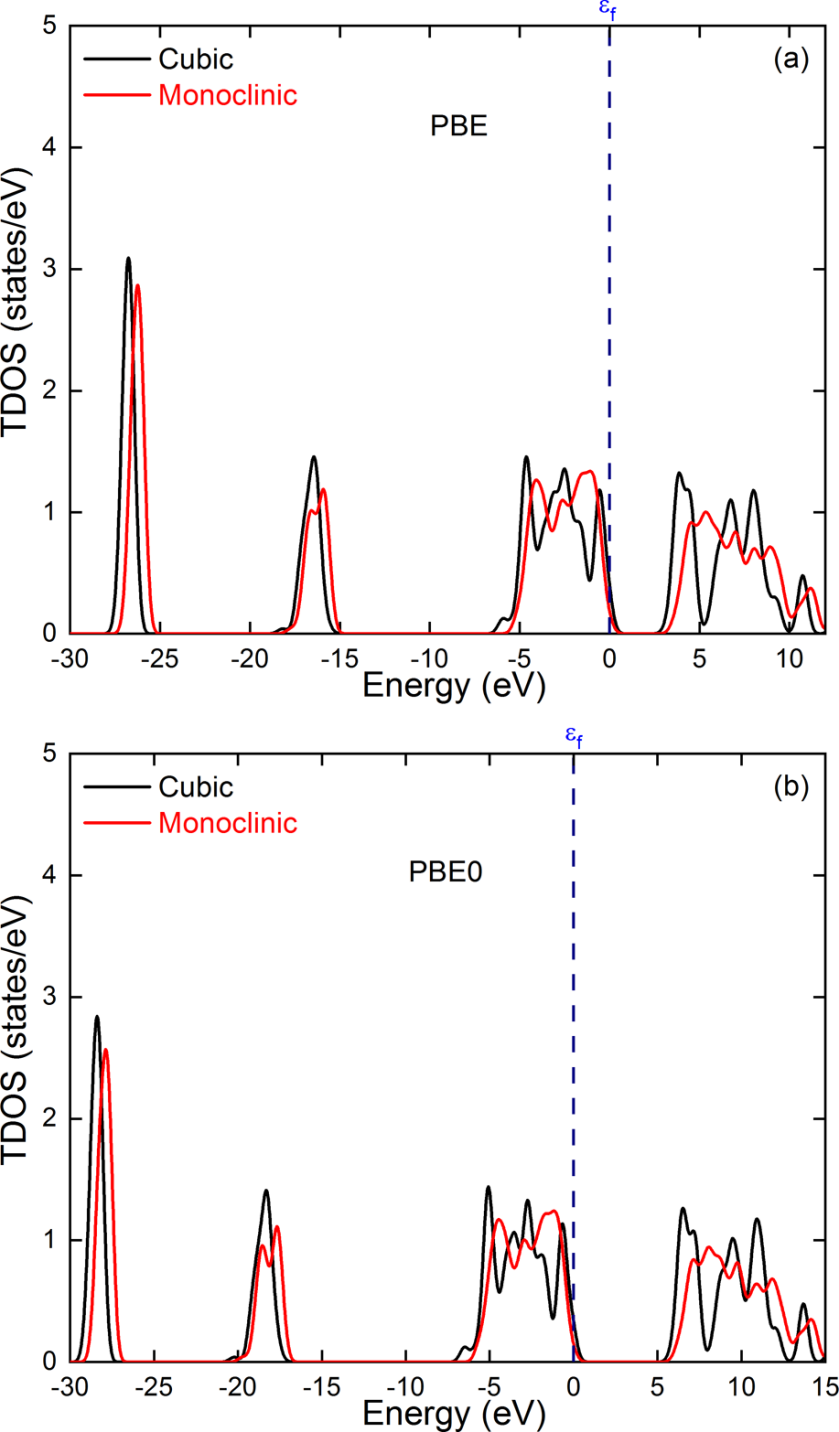
Total density of states (TDOS) obtained for the cubic and monoclinic polymorphs of zirconia using PBE (a) and PBE0 (b) approximations. The energy scale is referenced to the Fermi level.

**Figure 12:**
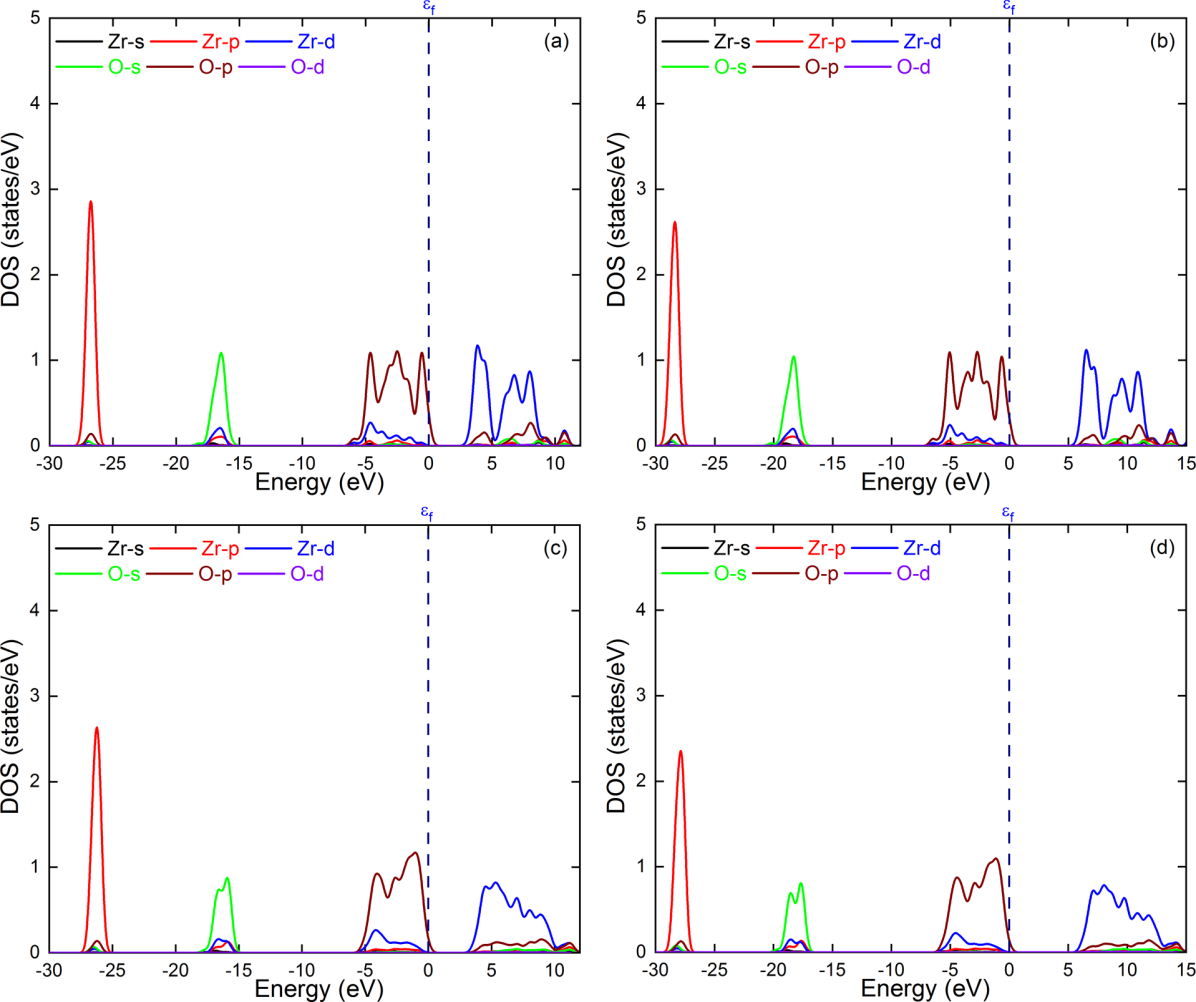
PDOS obtained for the cubic zirconia using the PBE (a) and (b) PBE0 functionals. Similarly, PDOS obtained for monoclinic polymorph using (c) PBE and (d) PBE0 functionals.

The TDOS and PDOS values calculated for the different saturation passivated NP models are presented in Figure 13. Contributions from H atoms are not plotted. It can be seen that the DOS of NPs and bulk materials share the same features, in terms of band positions and intensities. The DOS profiles of NPs are smoother and slightly broader than those of periodic systems, reflecting the structural disorder averaged over the 10 considered MD configurations. Interestingly, the shape of the valence bands of the NPs is asymmetric, with a bump on the higher-energy side, which more closely resembles the envelope of the monoclinic polymorph. This is observed for all model sizes and can be related to the monoclinic distortions present in the structure and described in the previous sections. Furthermore, the absence of defect levels in the band gap of the NPs reflects the effectiveness of our passivation procedure in eliminating surface defect states. It can be noted that the same calculations performed with the lowest passivation rate (16.66%) led to a defect level in the band gap and a narrowing of the band gap (see Figure S5 in the Supporting Information).

**Figure 13:**
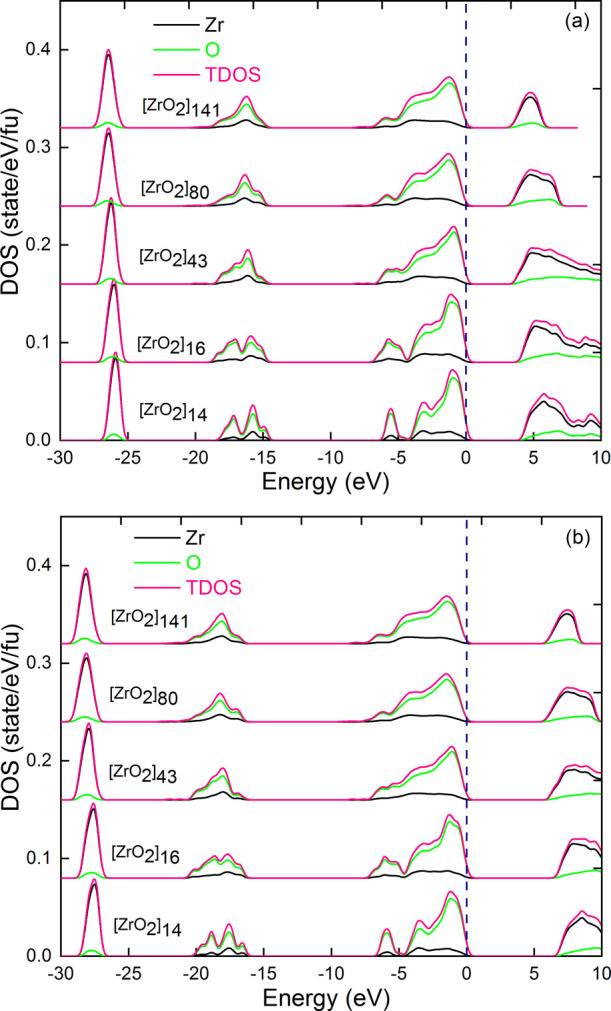
Partial and total density of states per formula units (fu) of the various saturation passivated NP models [ZrO_2_]_*n*_, *n* = 14, 16, 43, 80, and 141, using (a) PBE and (b) PBE0 approximations.

The band gaps calculated for the saturation passivated NP models (*E*_*g*_^NP^) are given in Table 1, together with their standard deviations. An increase in band gap values is observed as the NP diameter decreases, reflecting the appearance of a quantum confinement effect. The variations of the band gap as a function of NP size with respect to the bulk value of the cubic polymorph, Δ*E_g_* = *E*_*g*_^NP^ – *E*_*g*_^bulk^, are shown in Figure 14 for both PBE and PBE0 calculations. Reference is made to the cubic polymorph because, as shown above, the structure of NPs tends to be more cubic as their size increases. We can see that the trends obtained with PBE and PBE0 are very similar, with a tendency for PBE0 to give slightly smaller Δ*E_g_* values. This trend indicates that the quantum confinement effect in ZrO_2_ NPs is rather small, which can be explained by the large band gap of this material.^21^ Within the effective mass approximation, the size dependence of the quantum confinement effect for an ideal spherical NP in the strong confinement regime is given by

$$\Delta E_{g}=h^{2}/2\mu d^{2}$$ 1

where *h* is the Planck constant, *d* is the NP diameter, and μ is the reduced exciton mass, given by μ = 1/*m*_e_^*^ + 1/*m*_h_^*^ with *m*_e_^*^ and *m*_h_^*^ being the effective masses of the electron and hole, respectively.^6^ The band gap should thus decrease as *d* ^–2^ with increasing NP diameter. Comparing to our calculated data requires a good estimate of the effective masses of holes and electrons. Unfortunately, this is a challenging task for a wide-band-gap material like ZrO_2_, and few data exist in the literature. Using the (anisotropic) values calculated for the cubic polymorph in^63^, leads to an upper bound estimate of μ of around 0.8 electron mass units, a rather large value resulting from the material’s large band gap. The corresponding evolution using eq 1 is plotted in Figure 14. It is clear that despite the use of this μ upper bound, the band gap values obtained with our NP models are much lower and do not correspond to the variation given by eq 1. A reasonable fit of our data can be obtained using the common empirical expression^21,64–66^ Δ*E_g_* = β/*d* ^^α^^, with the value of the exponent α significantly greater than 2, around 2.65 (see Figure 14). The values found in the literature for many quantum dots with zinc blende or wurtzite structures (e.g., CdSe, CdS, CdTe, InP, GaAs, InAs, ZnO, etc.) are generally below 2.^21^ It should be noted, however, that in these cases the NP retains the ideal structure of the bulk material with a fully passivated surface. These observations certainly reflect the effect of the positional disorder present in the NP models, as well as the presence of incompletely saturated bonds at the NP surface.

**Table 1:** Calculated Band Gaps for the Zirconia Cubic Polymorph and for the NP Models Using PBE and PBE0 Exchange Correlation Functionals^a^

band gap (eV)		
models	PBE	PBE0
c-ZrO_2_ (bulk)	3.26	5.91
m-ZrO_2_ (bulk)	3.58	6.09
[ZrO_2_]_14_·8H_2_O	4.37 ± 0.05	6.98 ± 0.06
[ZrO_2_]_16_·12H_2_O	4.20 ± 0.07	6.85 ± 0.07
[ZrO_2_]_43_·24H_2_O	3.76 ± 0.04	6.26 ± 0.05
[ZrO_2_]_80_·26H_2_O	3.43 ± 0.04	5.90 ± 0.05
[ZrO_2_]_141_·54H_2_O	3.57 ± 0.06	6.06 ± 0.03

a Standard deviations obtained on the considered snapshots are also provided.

**Figure 14:**
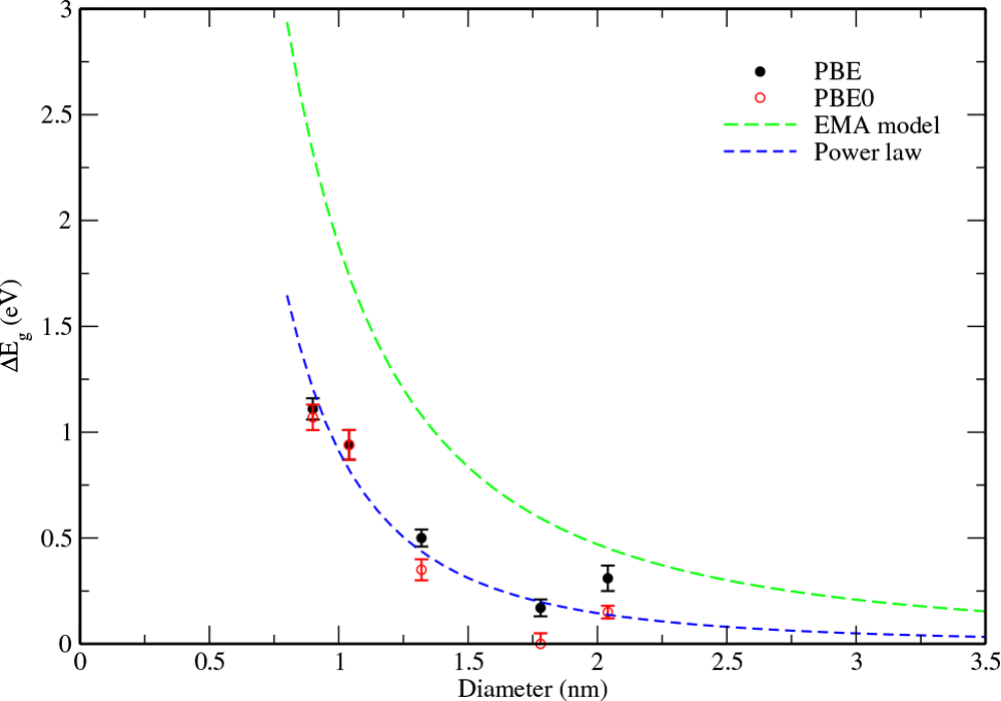
Band gap difference of saturation passivated NP models relative to that of the bulk material as a function of the NP size, computed using PBE and PBE0. The solid lines correspond to the band gap evolution following the effective mass approximation (Δ*E*_*g*_ ≈ *d* ^–2^). The dashed line is the evolution following the expression β/*d* ^α^ with α equal to 2.65.

#### 5.3.2 Core and Shell Atoms' Contribution to the Total DOS

By taking advantage of the analysis in Section 5.2.1, we here projected the DOS on the atoms belonging to the core and the surface regions of [ZrO_2_]_43_, [ZrO_2_]_80_, and [ZrO_2_]_141_ saturation passivated NP models, as shown in Figure 15. Overall, we observe that shell atoms give slightly larger and differently shaped DOS profiles compared with core atoms. This is particularly visible near the valence band maximum and the conduction band minimum, where the surface oxygen p-orbitals and zirconium d-orbitals induce large asymmetry on the higher- and lower-energy sides, respectively. In contrast, core atoms lead to DOS profiles closer to those of bulk phases. The slight valence-band broadening induced by the oxygen shell atoms (p-orbitals) results in a band gap decrease of around 0.15 eV compared with the band gap resulting from the central atoms alone. This contribution to the band gap decrease is not sufficient to explain the observed, albeit diminishing, differences seen in Figure 14 between values from NP models and those expected from the effective mass approximation model.

**Figure 15:**
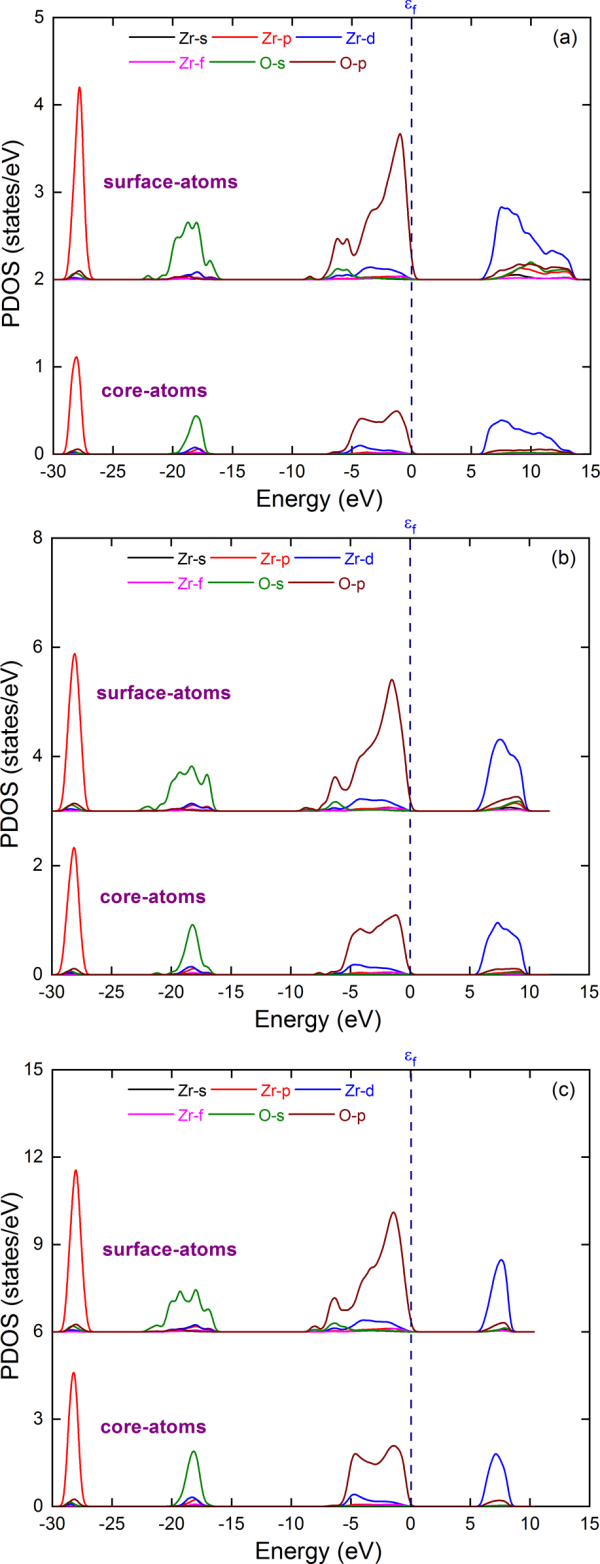
Decomposition of the partial density of states (PDOS) in terms of core and shell atoms for NP models [ZrO_2_]_43_ (a), [ZrO_2_]_80_ (b), and [ZrO_2_]_141_ (c) with a saturated passivation rate. PDOS of shell atoms are shifted vertically for clarity.

## 6 Conclusions

In this study, we investigated the relationships between the structural and electronic properties of [ZrO_2_]_*n*_ NP models as a function of their size (*n* = 14, 16, 43, 80, and 141) using ab initio molecular dynamics and DFT electronic structure calculations at the PBE and PBE0 levels of theory. A procedure, based on the use of water molecules and appropriate MD thermal annealing, has been developed to passivate dangling bonds on the surface of NPs up to a saturation rate. This procedure revealed that the rate of passivation has a significant influence on NP structure, and that NP models corresponding to saturated passivation exhibit the best structural characteristics, in close agreement with experiments. It was also found that the Zr–O bond length varies as a function of the position of Zr and O atoms from the core to the surface of the NPs, providing a descriptor capable of separating core and surface regions in ZrO_2_ NPs. A core–shell structure has been demonstrated for NP models as small as 1.3 nm, while for even smaller NPs, no separation between core and shell is possible. For the largest NP models, the core atoms show local environments closer to the cubic phase of zirconia, while the local structure of the atoms close to the surface shows a large similarity with the monoclinic phase. Finally, the study of electronic properties has shown that ZrO_2_ NPs exhibit very moderate quantum confinement effects. Moreover, the evolution of the band gap as a function of size does not correspond well to the *d* ^–2^ trend expected from the effective mass approximation model. Although part of this difference can be attributed to shell atoms, which induce a slight decrease in the band gap compared with the contribution of core atoms, it seems that it is the structural distortions present within the entire NP that must be taken into account to fully explain the differences observed.

## Supplementary Material



## Notes

Figures of pair distribution functions, density of states and molecular orbitals plots (PDF)
