# Supplementary material for: “Ultrasmall” ZrO2 Nanoparticles: Disentangling Core and Surface Contributions to Structural and Electronic Properties through First-Principles Modeling
Source: ACS Nanosci Au. 2025 Oct 13;6(1):54–67. doi: 10.1021/acsnanoscienceau.5c00088 (PMC12921586; doi:10.1021/acsnanoscienceau.5c00088)
Supplement: Supplementary file 1 [file ng5c00088_si_001.pdf]

"Ultrasmall" ZrO_2 Nanoparticles: Disentangling Core and Surface Contributions to Structural and Electronic Properties through First-Principles Modeling

Ravikant Kumar, Assil Bouzid*, Abid Berghout, Philippe Thomas, and Olivier Masson*

Institut de recherche sur la ceramiques (IRCER), CNRS UMR 7315, Universite de Limoges, Centre Europeen de la Ceramique, 12 rue Atlantis, 87068, France

E-mail: assil.bouzid@unilim.fr,olivier.masson@cnrs.fr

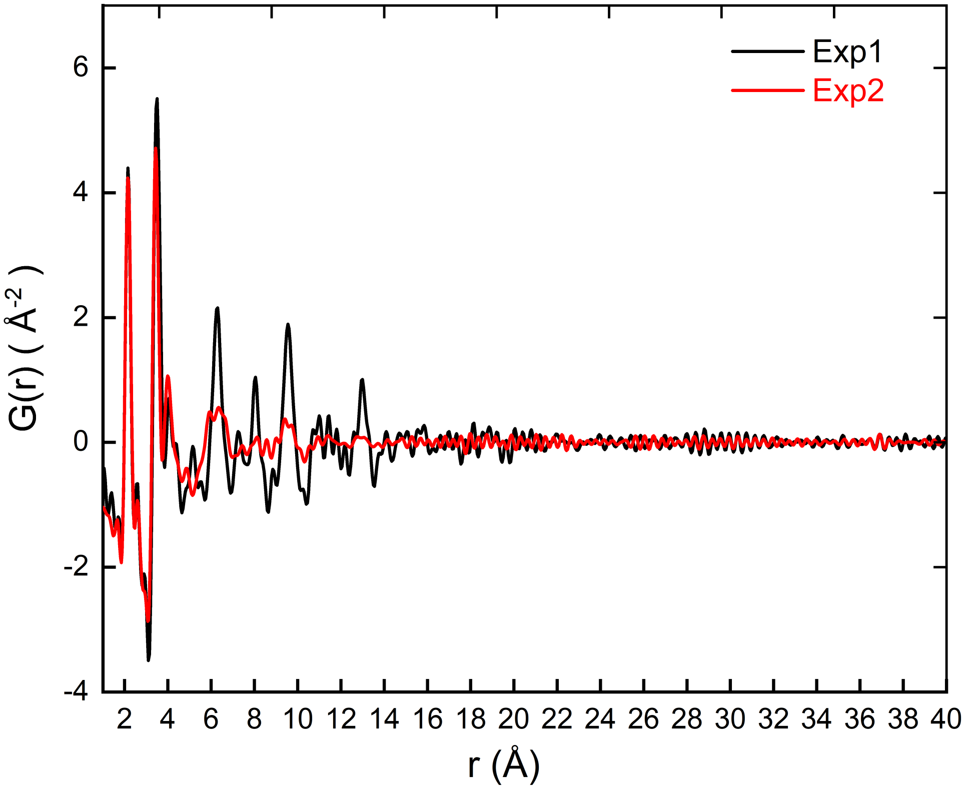

Figure SI 1: Experimental PDFs of sample Exp1 (black color) and sample Exp2 (red color). Data reproduced from Refs. [1,2]

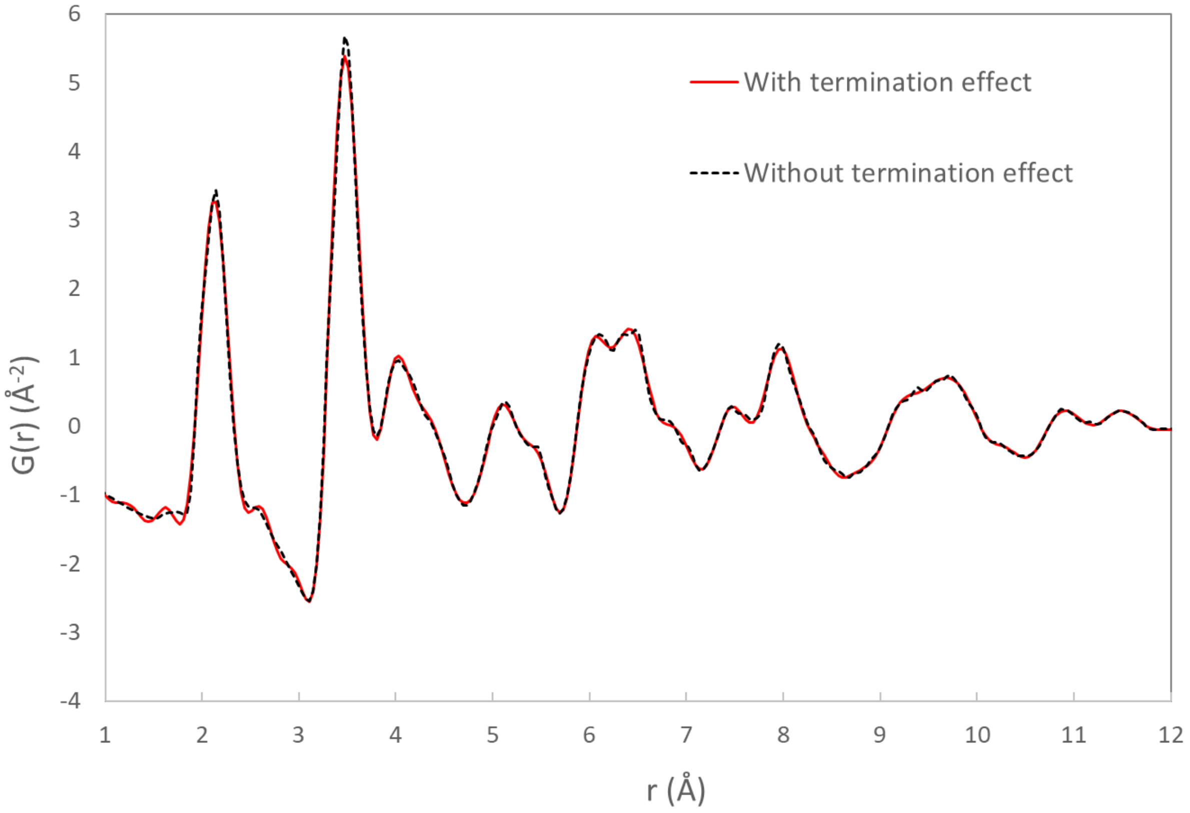

Figure SI 2: PDFs calculated from the $[ZrO_2]_{43}$-TT3 NP model with and without termination effect (i.e. with and without convolution with the $\sin(Q_{max}r)/(\pi r)$ function).

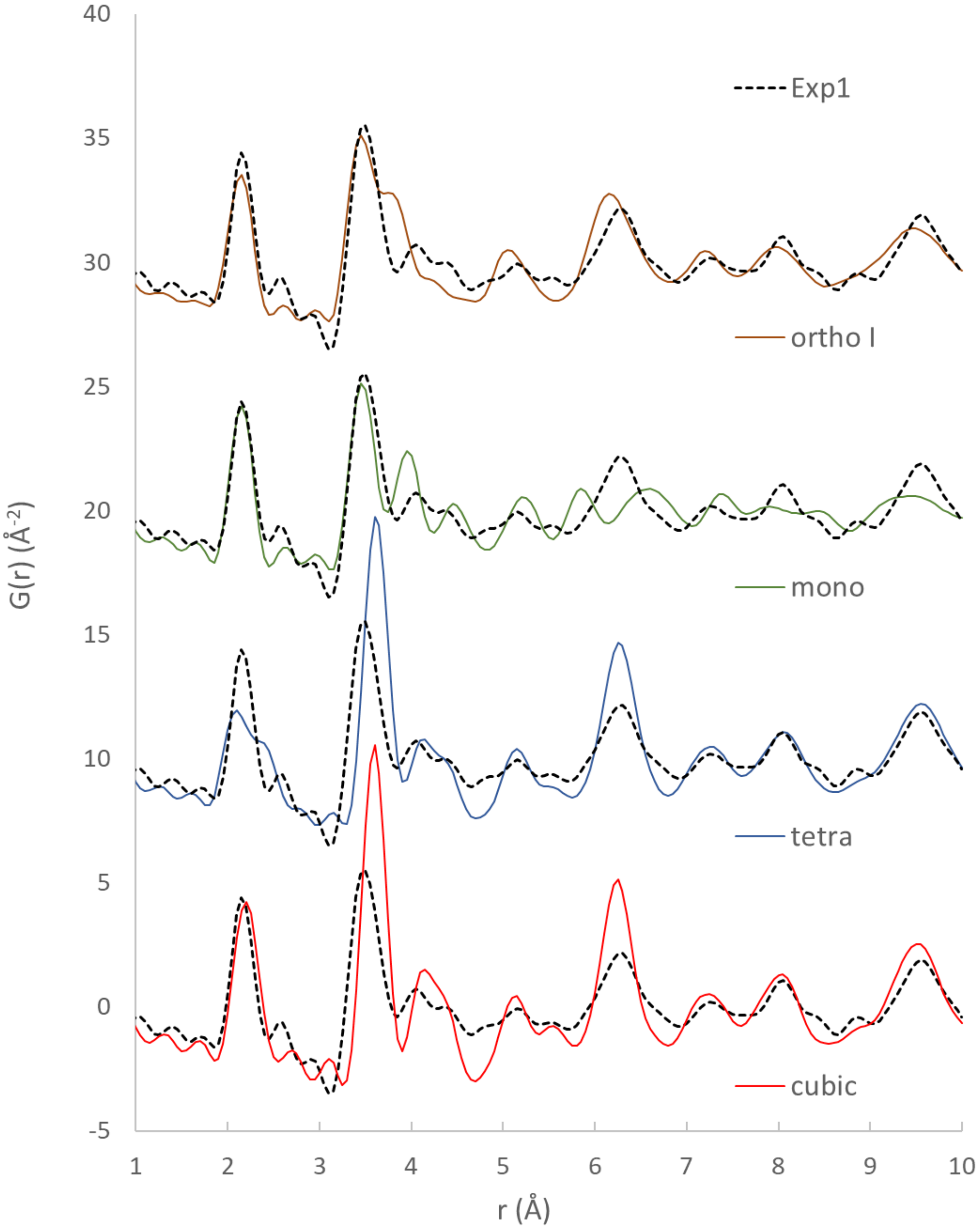

Figure SI 3: PDFs calculated from the four zirconia polymorphs deriving from the fluorite structure (from bottom to top: cubic, tetragonal, monoclinic and orthorhombic I) and compared to the experimental PDF of sample Exp1 (black dotted line). Calculations where performed using the PDFgui software[3] using the following parameters: NP size = 20 Å, $Q_{damp} = 0.038$, $Q_{max} = 17$ Å^{-1} and $Q_{broad} = 0.4$. The structural parameters of the monoclinic, tetragonal, cubic and orthorhombic I polymorphs were taken from references 4, 5, 6 and 7, respectively.

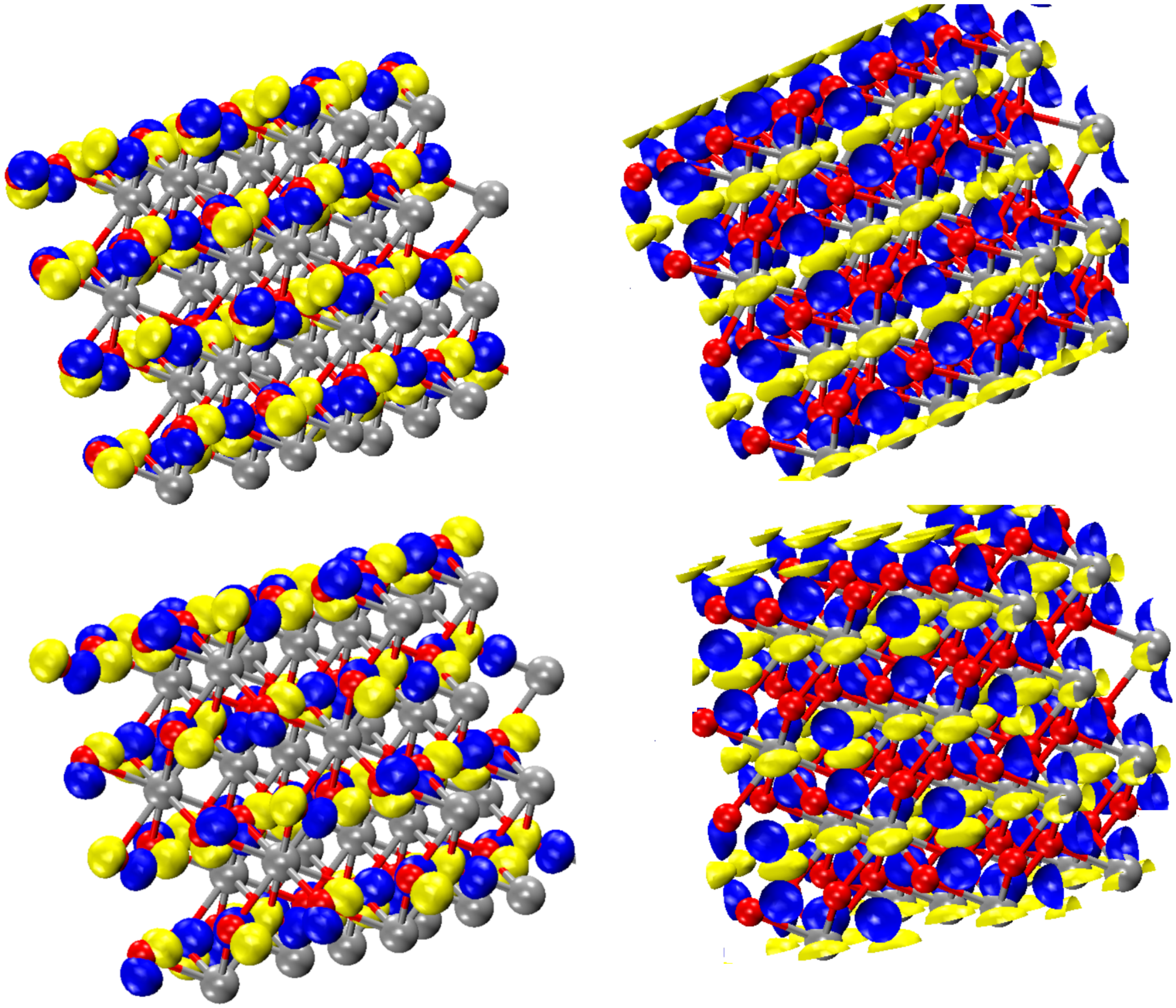

Figure SI 4: Molecular orbitals (MOs) plot for HOMO (left) and LUMO (right) of the cubic phase zirconia using PBE (top) and PBE0 (bottom) functionals by considering 2 % of isovalue.

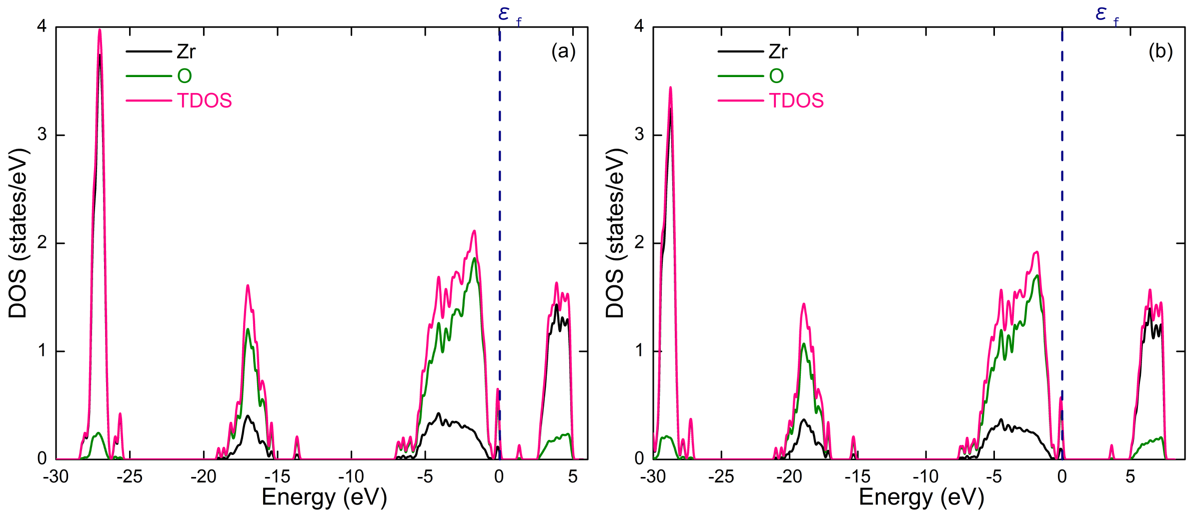

Figure SI 5: PDOS and TDOS for $[ZrO_2]_{43}$ with 16.66 % surface passivation using (a) PBE and (b) PBE0 functionals.

References

(1) Grena, R.; Masson, O.; Portal, L.; Remondiere, F.; Berghout, A.; Jouin, J.; Thomas, P. Stabilization effect of surface impurities on the structure of ultrasmall ZrO_2 nanoparticles: an ab-initio study. *The Journal of Physical Chemistry C* **2015**, *119*, 15618–15626.

(2) Gambe, J.; Jouin, J.; Remondiere, F.; Thomas, P.; Masson, O. Solvent effect in the nonaqueous synthesis of ZrO_2 nanoparticles under alkaline conditions. *Journal of Materials Science* **2020**, *55*, 2802–2814.

(3) Farrow, C. L.; Juhas, P.; Liu, J. W.; Bryndin, D.; Božin, E. S.; Bloch, J.; Proffen, T.; Billinge, S. J. L. PDFfit2 and PDFgui: computer programs for studying nanostructure in crystals. *Journal of Physics: Condensed Matter* **2007**, *19*, 335219.

(4) Howard, C. J.; Hill, R. J.; Reichert, B. E. Structures of ZrO_2 polymorphs at room temperature by high-resolution neutron powder diffraction. *Acta Crystallographica Section B* **1988**, *44*, 116–120.

(5) Lutterotti, L.; Scardi, P. Simultaneous structure and size–strain refinement by the Rietveld method. *Journal of Applied Crystallography* **1990**, *23*, 246–252.

(6) Katz, G. X-Ray Diffraction Powder Pattern of Metastable Cubic ZrO_2. *Am. Ceram, Soc.* **1971**, *54*, 531.

(7) Kudoh, Y.; Takeda, H.; Arashi, H. In situ determination of crystal structure for high pressure phase of ZrO2 using a diamond anvil and single crystal X-ray diffraction method. *Physics and Chemistry of Minerals* **1986**, *13*, 233–237.
